# Comparison of Superficial Mycosis Treatment using Butenafine and Bifonazole nitrate Clinical Efficacy

**DOI:** 10.5539/gjhs.v5n1p150

**Published:** 2012-11-11

**Authors:** Mohammed A. Abdul Bari

**Affiliations:** 1 Baghdad Collage of Pharmacy, Baghdad, Iraq

**Keywords:** butenafine, bifonazole, superficial mycosis

## Abstract

Superficial fungal infections are commonly encountered by the physician. And the continuously changing epidemiology of invasive fungal infections results in the need for an expanded armamentarium of antifungal therapies. This study was designed to evaluate the safety and efficacy of Butenafine (BTF) versus Bifonazole (BFZ) in the treatment of superficial mycosis in a randomized, double-blind, parallel-group trial. Of 96 patients, 48 applied (BTF) cream and 48 applied (BFZ) cream for 2 weeks to tinea versicolor, corporis and cruris treat, while tinea of feet & hands was treated for 4 weeks duration. Efficacy was assessed after the end of treatment and 2 weeks later. At the end of therapy, we find somewhat more patients using (BTF) than using (BFZ) had a mycologic cure ((BTF), 87.5%; (BFZ) 83.3%) and effective clinical response ((BTF), 91.7%; (BFZ), 83.3%). (BTF) provides rapid and persistent antifungal activity and symptom relief in patients with superficial mycosis during treatment. And patients continued to improve for at least 2 weeks after treatment. The Rates of mycologic cure and effective treatment with (BTF) were higher than with (BFZ) at cessation of treatment and 2 weeks later. However, no significant difference found between the two drugs (p> 0.05).

## 1. Introduction

Superficial fungal infections, including rare infections and commons skin disease confine to be limit to groups of patients or geographical areas. Superficial mycosis has been considered to be as major world public health problem and one of the most frequent diseases in human beings and animals (Burns et al., 2004; Kibbler et al., 1996; Midgley et al., 1997; Merlin et al., 1990). Hence; the superficial mycosis clinical treatment has been introduced by many of antifungal agents (Zarowny et al., 1995; Fredriksson, 1983; Kokoschka et al., 1986; McVie et al., 1986; Groll & Tragiannidis, 2010; Nolting et al., 1992; Reyes et al., 1998; Del Palacio et al., 1999). Nevertheless the increased use of such agents in recent years has caused the development of resistance to available drugs. For this reason, a constant effort toward the synthesis of new antifungal agents has been made in the last few years. In particular, the class of azoles (imidazole and Triazole derivatives) has supplied many effective antifungal drugs currently in clinical use, and newer azoles with expanded spectrum of activity are at the moment in continuous development (Han et al., 2011). Bifonazole (BFZ), an imidazole derivative, has a broad spectrum of action used currently for topical therapy of mycoses and dermatomycoses (Gupta & Sauder, 1994; European Pharmacopoeia Commission, 2001). Butenafine (BTF) is a synthetic benzylamine antifungal agent with fungicidal activity against susceptible organisms that have been approved in many countries worldwide (Georgopapadakou, 1998). The therapeutic efficacy of these two agents was compared in this study.

## 2. Materials and Methods

Our hospital received 96 patients with superficial mycosis disease complaints. Informed consent was obtained. We begin with complete medical history for each patient and record all data, including sex, age, and symptoms and signs of the disease. A physical examination included total body mass, weight and dermatological inspection. A clinical examination with standard laboratory tests was also carried out at inclusion and repeated after the end of treatment and 2 weeks later. In addition, vascular disease, particularly diabetes and other medical condition’s history are record. Hence, any patients received antibiotic or antifungal treatment for previous 90 days are excluded.

For this study 96-subjects with superficial skin mycosis by carry out KOH and fungal cultures positive randomized, parallel group; double-blind. The study group (subjects, including female 54 Male 42, age 14~72; duration of disease 3~10 months) divided into two treatment groups.

Of 96 patients, twice daily for 2 weeks each 48 applied (BTF) hydrochloride 1% and 48 applied (BFZ) 1% cream to the affected area to treat versicolor, corporis and cruris tinea and tinea of feet and hands need 4 weeks treatment. Efficacy was assessed after the end of treatment and 2 weeks late.

In this paper, the skin scraping by curetting of the affected webs was used for mycological study. For each patient, the Potas-sium hydroxide preparation was performed and Sabouraud dext-rose agar (SDA) was used for all specimens. Firstly, SDA with 0.005% chloram-phenicol to inhibit bacterial contamination and 0.05% cycloheximide to inhibit contaminating fungi were used for selective isolation of dermatophytes. Secondly, SDA without cycloheximide used to grow non-dermatophyte fungi that are commonly considered saprophytes or contaminants. With the aid of a straight needle, small fragments of skin were placed on the surface of the agar in Petri’s dishes containing 40 mL of culture medium and pressed onto the surface to make a good contact. The samples were adjusted into four different areas, or at least in two spots in the case of a scanty sample. Petri’s dishes were surrounded by paraffin seal to prevent dehydration and incubated at 30 °C for 4 weeks before being considered negative for fungus. Media were tested every 3–4 days for growth of fungus. Plates overgrown with contaminants growing from Petri dish edges were eliminated. Media were examined macroscopically and when applicable, the texture and surface color of the colony, as well as any pigment diffusing into the medium, were carefully noted and recorded. An adhesive tape strip was applied onto the surface of the colony, and then mounted in a drop of lactophenol cotton blue stain.

### 2.1 Drug Administration

The first group was treated with 1% (BTF) hydrochloride cream.

The second group was treated with 1% (BFZ) cream.

Both of them were treated by applying the cream twice daily for 2 weeks to treat tinea cruris, tinea corporis and Tinea versicolor. And 4 weeks for tinea of feet and hands

Where:

- 1% (BTF) hydrochloride cream

Brand Names: brand name: mai ke shu (Seaside Pharmaceutical Company)

- 1% (BFZ) cream

Brand Names: brand name: mei ke (Yongda drug manufactory)

### 2.2 Efficacy Criteria

A) Clinical Assessment

Beginning symptoms and signs for each Patient’s before, after treatment had been record and two-week later accord to 4 levels from zero to three depending on the degree of itching, redness, papules & pustules before as follows.

Zero=no, one=mild, two=moderate and three for server one.


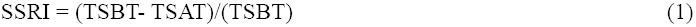


where

Symptom score reduces the index: SSRI

Total Score before Treatment: TSBT

Total Score after Treatment: TSAT

Cure: rash fade away; the symptoms disappear and microscopic examination of fungus and culture were negative, efficacy index 100%.

Obvious effect: rash markedly improved, symptoms relieved significantly; microscopic examination of fungus and culture were negative, the index effect 70%.

Improved: rash and symptoms were improved; microscopic examination of fungus and culture were negative or positive effect index 30% ~ 70%.

Not effective: skin rash did not disappear and, fungi examination and / or culture were positive, effective index less 30%.

B) Laboratory Assessment

Mycological Cure:

Clear: Both microscopically and fungal cultures were negative.

Not cleared: fungi examination and / or culture were positive.

C) Safety Criteria

Clinical checkup had been done before treatment, and patient’s blood, creatinine, urea, urine ALT, etc. then two weeks later all analysis done at cessation of treatment. All withdrawal treatment by laboratory abnormal parameters and vital signs also recorded.

D) Results Analysis

SPSS software was performed to analyse an intention-to-treat basis and to determine Quantitative variables such as means and standard relatively by class based on dichotomous variables as absolute or both deviations and categorical. The two tests (t-test and Mann–Whitney test) had been used to determine the significance of differences between mean values of two continuous variables, and to determine non-parametric distribution respectively. ANOVA as one-way analysis of variance was employed for comparison of several groups means and to determine the means significant differences between the two groups. In addition, multiple regression analysis was used to predictor the best diagnosis as a dependent variable. P < 0.05 was considered significant.

## 3. Result

### 3.1 Clinical Effectiveness

There was high efficiency noticed for both drugs after end of treatment and 2 weeks later as shown in Table 1 and Table 2.

**Table 1:** The effectiveness of clinical at of treatment

Group	cases	Cured	obvious effectiveness	Improved	efficiency
BTF group	48	12	32	4	91.7%
BFZ group	48	7	34	7	83.3%

Significant difference was p> 0.05 for the two groups

**Table 2:** Clinical effectiveness 2 weeks after stooping treatment

Group	cases	Cured	obvious effectiveness	Improved	efficiency
BTF group	48	36	9	3	93.7%
BFZ group	48	33	11	4	91.7%

Also no significant difference found by comparing the two groups there was p> 0.05

### 3.2 Mycological Cure

High clearance rate is also noticed for both drugs after end of treatment and 2 weeks later as shown in Table 3 and Table 4.

**Table 3:** Mycological cure at end of treatment

Group	cases	cleared	clearance rate
BTF group	48	42	87.5%
BFZ group	48	40	83.3%

There wasn’t any significant difference (p> 0.05)

**Table 4:** Mycological cure weeks after stooping treatment

Group	cases	cleared	clearance rate
BTF group	48	46	95.8%
BFZ group	48	44	91.7%

Similarly, there wasn’t any significant difference (p> 0.05)

There was not any serious adverse effects.

For (BTF) cream there was no noticeable adverse reaction; also for (BFZ) cream apart from local skin erythema and mild itching which require no treatment. Finally, no abnormality was found by laboratory examination for the two group’s treatment.

## 4. Discussion

The progress in managing of fungal infections, the incidence increased and response rates remain insufficient and this may led to the development a new antifungal agent. Recent antifungal chemotherapy drugs show new advances in offer clinician’s fewer toxic alternatives and more effective at conventional therapy. BTF hydrochloride has similar action a synthetic benzylamine derivative mode to that of the allylamine class of antifungal drugs (Gerald & Mc Evoy, 2008).

The evaluation of randomized, double-blind, controlled trial for the safety and efficacy of 2 weeks, twice-daily treatment with (BTF) versus (BFZ) for each patient with versicolor, cruris, and corporis tinea; and 4 weeks to treat tinea of feet & hands. The final comparing the treatment of BTF group mycologic cure higher than a BFZ show group (87.5, vs. 83.3%, p> 0.05). Similarly, the mycologic cure rates at the 2 weeks follow-up were 95.8% vs. 91.7%, respectively (p >0.05). Clinical efficiencies were 91.7% for (BTF) group, compared to 83.3% for BFZ group in the (p>0.05). Then after 2 weeks clinical efficiency was 93.7% for (BTF) group, compared to 91.7% for BFZ group (p> 0.05). These results show that both (BFZ) and the significant advance for newer (BTF) treatment of fungal infections. Their expanded activity gives the clinician efficacious, safe, inexpensive and available more choices other than conventional treatment. Particularly, the optimal treatment of superficial mycosis still remains in the resistant cases with neither drug class offering an overwhelming advantage. Slightly higher rates of mycologic cure and effective treatment, (BTF) group offers a little advantages over (BFZ) group. The BTF main benefits are appeared as safety, ease of use, and offset by its current additional costs.

No serious side effects were noted with (BTF) except for mild itching and skin rash, but it was tolerable and no patient discontinued therapy due to an adverse event. In the adverse events for most commonly uncontrolled trials, associated with the use of (BTF) group 1% cream included erythema, contact dermatitis, itching, and irritation, for each occurring in less than 2% of patients (Syed et al., 1998).

## 5. Conclusion

In this paper, during patient treatment (at least 2 weeks) BTF provides rapid improving antifungal activity and symptom relief with superficial mycosis The Rates of mycologic cure and effective treatment with (BTF) were higher than with (BFZ) at cessation of treatment. However, statistically there was not any difference significant between the two drugs (p> 0.05).
